# Dental Caries among Certain Southeastern African Tribes

**Published:** 1919-12

**Authors:** Stanley Colyer


### DENTAL CARIES AMONG CERTAIN SOUTHEASTERN AFRICAN TRIBES

BY STANLEY COLYER, M.D., L.D.S.

In the Transkeian territories the natives may be said to have been given over to the missionaries and other educationalists for many years, so that although the territories are a native reserve, the number who wear European dress and who speak the English language is remarkable. There remains, however, a fair residuum who reject both the advantages of education and the attractions of the white man's garments, and continue to dress in their picturesque national costume, and follow, to a large extent, many of their old customs. These latter are contumaciously called the "Raw Kaffir," but more correctly the "Red Kaffir," on account of their habit of dyeing their blankets and clothes a reddish ochre color. Though called "raw," the name is, generally speaking, a misnomer, as most of them have dealings with the European stores both for articles of attire and certain groceries. A few of the natives of these territories are exceptionally rich, some being masters of hundreds and even thousands of pounds, the capital being represented in cattle, sheep, goats and grain. They belong to the Bantu section of the human race, just as do the Central Africans, but differ from them both in appearance and habits. For many years real war may be said to have been absent from these parts—the wild animals, both buck and the fierce and dangerous carnivora, have been killed out—a motherly government has carefully protected them; teachers have endeavored with doubtful success, to improve their minds; while traders have sold to them without any sentimental regard whatsoever for their well-being. In losing their natural occupations they have lost much of their hardiness, but they have retained suspiciousness and cunning, characters which seem to be inseparable from the Bantu nature.

From a dental point of view they are instructive, more especially to one who has had the opportunity of studying their more primitive and harder brethren in Central Africa. Here, for the first time in my experience, we find dental caries like that of civilized man, and by that I mean interstitial and fissure caries. Natives may be found with every tooth, save, perhaps, a few isolated ones in their jaws, decayed and level with their gums. Caries of the incisors is comparatively common. Children are as frequently affected as their elders. Rickets is beginning to appear and oral sepsis, primarily due to decayed teeth, is becoming an aetiological asset, especially in children. When we turn to the question of pyorrhea, we find that although fulminating pyorrhea—and by that I mean cases in which a few teeth only are affected, but in which the disease is rapid and advanced loose teeth, deep pockets, and a free flow of pus being present—may be seen occasionally; general periodontal trouble is comparatively rare. I have examined many old men of over fifty, in the government gaol, and, as far as I can remember, I have not seen one with advanced general periodontitis, though such cases may occasionally be seen among some of the rich, old men.

When I compare my experience here with my experience in Central Africa, I find that just the opposite conditions hold; that whereas dental caries is common here, it is uncommon there; that whereas pyorrhea is common there, it is uncommon here. Such a contrast as this seems to hold out a hope that by a careful consideration of the two environments we may find the causes both of caries and of pyorrhea. In my opinion the deductions that can be drawn from such a consideration are strongly in favor of the views that I put forward many years ago (1904), but it is not my intention to restate the many reasons I have for holding them, nor do I here intend dealing in detail with the subject of pyorrhea. Rather do I wish to continue the record of my dental observations on African natives, which it has been my good fortune to have the opportunity of making.

The food of the Transkeian natives may be placed in five classes:

1. Grain:
   - a. Mealies (umbona).
   - b. Kaffir corn, brown and white (amazimba).
   - c. Wheat. A recent introduction and but little grown.
   - d. Rice. Imported and but little used.

2. Vegetables:
   - a. Potatoes. The European kind only.
   - b. Pumpkins.
   - c. Beans and peas. The seeds only eaten.
   - d. Certain roots, consumed by Raw Kaffirs only.

3. Meat and Flesh Foods:
   - a. Oxen, sheep and pigs, chickens and other birds.
   - b. Caterpillars and certain insects in season. Consumed by the Raw Kaffirs only.

4. Savories and Relish Foods:
   a. Sugar.
   b. Salt.
   c. Pepper.
   d. Curry powder.
   e. Leaves of potatoes, pumpkins and other plants.

5. Drinks:
   a. Fermented and unfermented Kaffir beer.
   b. Milk. Generally as sour or curdled milk.
   c. Tea and coffee.
   d. Water.

Grain:—The staple foods are mealies and Kaffir corn. All other foods are subsidiary. The methods of preparation that the natives under consideration adopt differ in certain essentials from the methods adopted in Central Africa, the chief being that the bran or husk of the grain is not so carefully excluded. As both mealies and Kaffir corn are prepared in much the same ways, the following descriptions will apply to each. The grain is bruised, broken and crushed in a wooden mortar. Depending on the taste and energy of the worker, a fine or coarse powder is produced. In cases where the grain in only coarsely ground (or more correctly, broken) it is subsequently washed before cooking. By this process the larger pieces of thin husk float apart and are removed. In finer ground grain, the powder is poured from basin to basin in the air, and by this means the lighter bran is blown away. In some cases the coarse powder is further ground between stones, and thus a very finely-divided powder is produced. A certain amount of machine-ground grain is now purchased at the stores; it is not winnowed before being sold. The powder, whether of fine or coarse, is mixed with water and boiled until cooked. It is then mixed with some flavoring material, such as pumpkin, beans, salt, sugar, etc., and is served as a stiff mass and eaten cold.

WHEAT:—This grain is of recent introduction. Those few natives who eat it grind it like other grain, and make a leavened mass out of it which may be called bread (isonka). Mealie meal is sometimes mixed with the wheat.

VEGETABLES:—These require but little notice. They are boiled until soft and are then mixed with the porridge of mealie meal or of Kaffir corn.

MEAT AND OTHER FLESH FOODS:—By certain natives meat is consumed in enormous quantities, but, to the main mass of people, it must be regarded as a luxury. It is eaten raw, roasted, boiled, or rotten—depending upon the taste and status of the individual. Chickens and other birds are consumed in the same way. Caterpillars and other insects are seasonal and are said to be reserved for the Raw Kaffir—but I doubt it.

SAVORIES AND RELISH FOODS:—The various leaves are boiled. Pumpkin, though generally boiled and mixed like the leaves with porridge, may be roasted and eaten alone. Salt:—I know of no local source of this condiment, so that in the old days it was probably obtained by burning grasses. It is mixed with the food in the same way as in Europe. Large quantities are sold at the stores. Sugar: —This is an introduction of recent years. The local climate, being temperate, will not support, generally speaking, the sugar cane. There are a few places where, so I am told, it is grown, though I have traveled much through the surrounding country and have not seen it. The natives use sugar in much the same way as we do. When we consider that it is only in recent years that sugar, as such, has been introduced to the natives, we are justified in saying that, in some instances, they consume enormous quantities. Thus my interpreter's family, which consists of six members, consumes six pounds a week, and I was recently informed by a trader that he often sold four packets a day (about 280 lbs.). The native mixes it sometimes with his porridge, sometimes eats it raw, though his chief use for it is to flavor his tea and coffee. To a cup of tea or coffee he puts two and sometimes three spoonsful. He likes his tea and coffee syrupy, as anyone who has received his hospitality knows, and he drinks it three or four times a day. Some native women, and a few men, often eat sugar whole; mothers may not only give it to their children by day, but will pacify a fretting child at night with it. In connection with sugar I may mention "sweets." These, so I am told, can hardly be said to have "caught on," though they are sold in fair quantities. Generally, however, they pass to the native in the shape of a present (basela) at the end of a business transaction—a handful being passed over the counter.

DRINKS:—These do not closely concern us. Kaffir beer is made of a mixture of mealies and Kaffir corn and contains either none or much alcohol, according to the time it is allowed to mature. Sour milk (amaasi) is more or less a luxury. I do not think it is responsible for dental caries in these natives. Tea and coffee I have already dealt with. Water requires no notice.

It would take too much space to enter into a detailed comparison of the food, say of the Barotzi of Central Africa, which offers a great contrast, and the food of the natives under consideration. I must refer anyone interested in the subject to my paper in the *Dental Record* for January, 1916. It will suffice here to say that the main differences in the respective foods are: that, whereas, the Barotzi very carefully winnow their crushed grain, and eat, in addition, as their main article of diet, a root or corn, known as cassava (mwanga), and their food forms (when prepared of cassava) a tenacious stodgy, elastic, and rather sticky mass, these natives do not, as a rule, crush their grain so fine, are unaware of the existence of cassava, and their food forms, when prepared, a hardish, rather fragile and rough mass. Among the Transkeian natives least tainted by European customs, such food, with the addition of local savories, as leaves, pumpkins, beans, and purchased salt, is practically their only diet. Old men may be seen with teeth well worn down and free of caries and with gums generally free of pyorrhea. In corresponding old men of Barotzi the same holds good with regard to caries, but pyorrhea is practically universal. The cassava of the Barotzi is a sticky food and hangs about the mouth, whereas the porridge of the Transkeians exerts, if anything, a natural, cleansing action; and yet in neither case does caries supervene. It must be added that the old Transkeian natives cleaned their teeth with their fingers and a little fine sand or ash from a fire. This custom is, unfortunately, dying out, and I can maintain with considerable certainty that you never see a really clean mouth unless some form of cleansing is adopted. Such was the old native. To-day, though some of the ''Red Kaffirs'' live in the same way, the more up-to-date natives have taken over some European customs and altered their food in many ways. Thus, they may take their porridge of a looser consistency, eating it with a spoon, they may flavor it with sugar, their tea and coffee they drink as syrup, and yet, so far as I can judge, so long as they do not take sugar, or sugary substances, after their last meal at night, they remain free from caries, whether they clean their teeth or not. The really important result of cleansing the teeth as carried out by the natives being to prevent pyorrhea.

It is not possible in this district to separate satisfactorily the influence of bread from that of sugar, but the influence of sugar can be easily separated from that of bread, for these reasons: that wherever bread is consumed there also is sugar consumed (chiefly among women servants), but hundreds may be found who practically have never tasted bread and to whom sugar is a daily food. I see children from all parts of the district who have never, or scarcely ever, tasted bread, and whose teeth, in many cases, are in as appalling a condition as one may see in some slum children at home. Never once have I seen such a child to whom sugar is not frequently given and sweets sometimes. You may search through all the untouched villages of Central Africa for a similar example, and I guarantee that you will not find one.

In order that I may not mislead the reader he must bear in mind that the proportion of these natives at present affected with caries, as compared with people of civilized countries, is still small. I am, unfortunately, unable to produce any statistics, as I have found it impossible, for many reasons, to collect any that would be of scientific value. My experience is that the women are more affected than the men (and here I may say that almost all the men are inveterate smokers); that the worst cases I see are among women servants and the wives and children of the wealthier classes; that a large proportion of the children who are brought to my surgery, perhaps ten to fifteen per cent. are affected to a greater or less extent; and that the disease is rare among the Red Kaffirs.

To one who has studied this disease among civilized people only, it may be difficult, perhaps impossible, to estimate in relation to its causation, the relative importance of the various foodstuffs and of their methods of preparation; but to another who has been trained both in dental and general pathology, who has had not only European experience, but the opportunity of observing African natives in various parts of their country and in varying stages of their movement towards European civilization, there can be little doubt. As a result of my experience I have no hesitation in saying that the fermentable sugars, the mono- and di-saccharids are the important factor, the cause par excellence; that the soluble fermentable polysaccharids (soluble starches) are a just perceptible cause and that soft food per se is harmless unless mixed with easily fermentable carbohydrates.—*Dental Record*, November, 1919.

